# Prospective Associations of the Short Form Health Survey Vitality Scale and Changes in Body Mass Index and Obesity Status

**DOI:** 10.1155/2018/3671953

**Published:** 2018-08-01

**Authors:** Cathrine Lawaetz Wimmelmann, Emilie Rune Hegelund, Anna Paldam Folker, Emilie Just-Østergaard, Merete Osler, Erik Lykke Mortensen, Trine Flensborg-Madsen

**Affiliations:** ^1^Section of Environmental Health, Department of Public Health, University of Copenhagen, Øster Farimagsgade 5A, 1353 Copenhagen K, Denmark; ^2^Center for Healthy Aging, Faculty of Health and Medical Sciences, University of Copenhagen, Copenhagen, Denmark; ^3^National Institute of Public Health, University of Southern Denmark, Øster Farimagsgade 5A, 1353 Copenhagen K, Denmark; ^4^Research Center for Prevention and Health, Glostrup Hospital, Glostrup, Denmark; ^5^Danish Aging Research Center, University of Aarhus, Aarhus, Denmark; ^6^Danish Aging Research Center, University of Southern Denmark, Odense, Denmark; ^7^Danish Aging Research Center, University of Copenhagen, Copenhagen, Denmark; ^8^Section of Social Medicine, Department of Public Health, University of Copenhagen, Øster Farimagsgade 5A, 1353 Copenhagen K, Denmark

## Abstract

**Objectives:**

The objectives of the current study were to prospectively investigate the predictive value of the vitality scale of the Short Form Health Survey for changes in body mass index and development of obesity.

**Methods:**

The study population comprised 2864 (81.5%) men and 648 (18.5%) women from the Metropolit Project and the Danish Longitudinal Study on Work, Unemployment and Health, who participated in a follow-up examination in 2009–2011 corresponding to a follow-up period of 3–7 years. Associations of vitality with body mass index and obesity were investigated separately for men and women in linear and logistic regression models adjusting for age, baseline body mass index, education, physical activity, smoking, and obesity-related diseases.

**Results:**

Vitality was significantly associated with change in body mass index among men (*p* < 0.001) and women (*p* < 0.05) gaining weight after adjusting for age, baseline body mass index, education, physical activity, smoking, and obesity-related diseases. No significant associations of vitality with BMI change were observed among individuals maintaining or losing weight during the follow-up period. Furthermore, vitality significantly predicted development of obesity among women.

**Conclusion:**

The study indicates that vitality is of predictive value for increases in BMI over time among individuals gaining weight and may further predict the development of obesity among women. This identification of poor vitality as a potential risk indicator for weight gain and development of obesity may be beneficial in clinical practice.

## 1. Introduction

Obesity, defined as a body mass index (BMI) ≥30 kg/m^2^, is a major public health problem [1] and is associated with various medical comorbidities such as type 2 diabetes, cardiovascular disease, and stroke [2]. In addition to the medical comorbidities, a growing body of research has investigated the psychological and social correlates of obesity. Especially, health-related quality of life (HRQOL) has received attention [3–5]. HRQOL has been defined as a multidimensional construct comprising physical, emotional, and social well-being, which reflects an individual's subjective evaluation and reaction to health or illness [4]. The Short Form Health Survey (SF-36) [6] is a widely used generic measure of HRQOL comprising eight subscales that can be divided into a physical and a mental HRQOL component. By applying generic measures of HRQOL such as the SF-36 [6], cross-sectional studies have shown that HRQOL varies with BMI [7, 8]. Most consistently, individuals with obesity seem to have lower HRQOL [9, 10]. Furthermore, cohort studies have shown decreases in HRQOL over time in overweight individuals and in individuals with obesity with the largest reductions observed in the physical domains of HRQOL [11, 12]. Finally, weight loss has been associated with increases in several HRQOL domains especially vitality, general health perception, and physical role functioning [13, 14].

However, BMI and obesity may not merely predict decreases in HRQOL but may also be a consequence of HRQOL. Thus, HRQOL may be a predictor of BMI and obesity, but until now, only one study has investigated whether HRQOL predicts weight changes over time. This study [11] indicated a bidirectional relationship between HRQOL and obesity. Interestingly, individuals in the lowest tertile of the HRQOL domains related to the mental component gained significantly more weight over the five-year follow-up period than individuals in the highest tertile. That is, where previous research has emphasized the impact of obesity on the physical aspects of HRQOL [10, 12], this study suggested that mental aspects of HRQOL is of importance for weight changes over time.

The SF-36 vitality scale is a subscale included in the mental component of the SF-36 and assesses different aspects of fatigue. This scale has been suggested as the best of the eight SF-36 subscales at discriminating between levels of health [6, 15], and it has previously been associated with several medical conditions, including cardiovascular disease and mortality risk [15, 16], suggesting that somatic aspects of health is also captured by the SF-36 vitality scale.

As knowledge about the possible effects of HRQOL on weight status is extremely scarce, the aim of the current study was to investigate the predictive value of the SF-36 vitality scale for both changes in BMI and development of obesity. We set out to examine this question in a longitudinal study design using two large Danish cohorts that were examined in 2004 and 2006, respectively, and included a follow-up study of these cohorts 3 to 7 years later. As sociodemographic factors, lifestyle, and health factors consistently have been associated with both HRQOL [17, 18] and weight status [19, 20], the effects of such factors on the associations between SF-36 vitality and BMI and obesity will be included in the analyses of this relation.

## 2. Methods

### 2.1. Study Cohorts and Baseline Data

#### 2.1.1. The Metropolit 1953 Danish Male Birth Cohort (MP)

The Metropolit 1953 Danish Male Birth Cohort [21] comprises 11532 men born in 1953 in the metropolitan area of Copenhagen who were alive and living in Denmark in 1968. In 2004, a questionnaire was sent to all members of the cohort who had a postal address in Denmark, assessing different aspects of health, social position, and health behavior. A total of 6292 men aged 51 years completed the questionnaire which included the SF-36 vitality scale and information on self-reported height and weight.

#### 2.1.2. The Danish Longitudinal Study on Work, Unemployment and Health (DALWUH)

The Danish Longitudinal Study on Work, Unemployment and Health (DALWUH) [22] is a random sample of 10% of the Danish population born in 1949 and 1959. Questionnaire data concerning health, social relations, psychosocial factors, and work environment were collected in 2000 and again in 2006. A total of 6151 men and women aged 47 and 57 years participated in the 2006 follow-up, which included the SF-36 vitality scale and information on self-reported height and weight.

#### 2.1.3. The Copenhagen Aging and Midlife Biobank (CAMB)

**Figure 1:**
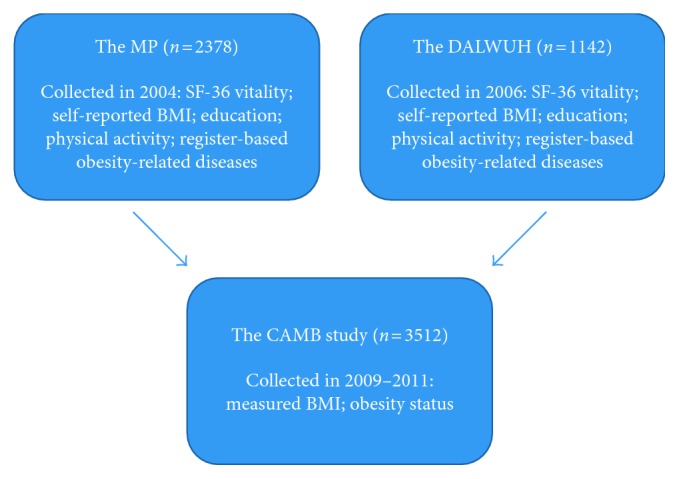
Baseline cohorts and follow-up sample. MP,  Metropolit cohort; DALWUH, the Danish Longitudinal Study on Work, Unemployment and Health; CAMB,  Copenhagen Aging and Midlife Biobank.

In 2009–2011, members from the MP and the DALWUH were invited to participate in the Copenhagen Aging and Midlife Biobank (CAMB) [23]. Participants in the CAMB completed a questionnaire assessing health, lifestyle behaviors, social position, and social relations. In addition, they participated in a clinical examination including an assessment of BMI and a blood draw. Written consent was obtained from all participants prior to examination. Complete information on the SF-36 vitality scale at baseline and BMI at baseline and follow-up were available for 2378 men from the MP and 1142 men and women from the DALWUH. To avoid outliers with regard to BMI, eight men were eliminated from the analyses due to an extreme BMI change from baseline to the CAMB examination. Thus, the final study sample for the current study comprised 3512 participants with a mean age of 51.6 years (SD: 3.0 years) (Figure 1).

The study protocols of the described studies were approved by local institutional review committees prior to study initiation of the studies.

## 3. Measures/Variables

### 3.1. Outcomes

#### 3.1.1. Body Mass Index and Obesity

BMI was calculated as weight (kg)/height (m)^2^. At follow-up, height and weight were measured at a clinical examination [23], while BMI at baseline was based on self-reported height and weight. BMI change was estimated as “follow-up BMI−baseline BMI” and used as a continuous outcome variable in linear regression analyses.

Obesity was constructed as a binary variable defining participants with a BMI ≥30 kg/m^2^ as having obesity and participants with a BMI <30 kg/m^2^ as not having obesity.

### 3.2. Predictor

#### 3.2.1. SF-36 Vitality

Vitality was assessed using the vitality scale of the Short Form Health Survey (SF-36) [6], which consists of four items evaluating the vitality domain of HRQOL by questions on tiredness and energy level. For instance, one question was “*How much time during the past 4 weeks did you feel full of pep?*” Answers ranged from 1 (“All of the time”) to 6 (“None of the time”). Raw scores from the SF-36 vitality scale were transformed to scores ranging from 0 to 100 with 100 indicating the highest level of vitality. Based on the tertiles of the SF-36 vitality scale scores, a categorical variable with three categories corresponding to high, medium, and low vitality was constructed and used in the main analyses.

### 3.3. Covariates

Variables that have previously been shown to affect both HRQOL and weight status were considered as covariates.

#### 3.3.1. Educational Level

Educational level provides information about the length and the level of the participants' vocational training at baseline assessed on six categories ranging from 1 (“No vocational training”) to 6 (“Higher level education >4 years”). Based on this information, three categories were derived corresponding to low, medium, and high level of education.

#### 3.3.2. Leisure Time Physical Activity

“Leisure time physical activity” was self-reported at baseline. Participants in the MP were asked which of the following four statements best described their general level of leisure time physical activity: hard exercise at elite level several times a week; exercise or heavy domestic work at least 4 hours a week; easy physical activity such as walking and cycling at least 4 hours a week; or sedentary activity such as reading and television watching. Participants in the DALWUH were asked to report the average number of hours per week they were physically active. Answers ranged from 1 (“0 hours”) to 6 (“>7 hours”). Information about physical activity level in the two cohorts was combined in a categorical variable. Based on the distribution of answers on the cohort-specific physical activity items, three categories were constructed corresponding to low, medium, and high physical activity level.

#### 3.3.3. Smoking

A question about current smoking status was administered to participants in the two cohorts. All participants were asked whether they were currently smoking on a daily basis; currently smoking but not on a daily basis; had stopped smoking; or never had smoked. Based on this information, a binary variable was constructed with participants who reported that they were currently smoking either daily or regularly categorized as “smokers” and participants who reported to have stopped smoking or never had smoked categorized as “nonsmokers.”

#### 3.3.4. Obesity-Related Disorders

The Danish National Patient Registry was used to identify participants who had received one or more of the following obesity-related diagnoses prior to the baseline assessments in 2004 (the MP) and 2006 (the DALWUH): type 2 diabetes mellitus (ICD-8 code: 250; ICD-10 code: E11), essential hypertension (ICD-8 codes: 400, 401; ICD-10 code: I10), ischemic heart disease (ICD-8 codes: 410–413; ICD-10 codes: I20, I21, I24, I25), or other hypothyroidism (ICD-8 code: 242; ICD-10 code: E03). These conditions were selected as they are frequent among elderly persons and have been included in prior studies of HRQOL and BMI [11]. Four variables corresponding to the four conditions were constructed and scored such that 0 reflected “no diagnosis” and 1 reflected “diagnosis.”

#### 3.3.5. Statistical Analysis

Descriptive statistics were used to analyze participant characteristics. Differences between the two cohorts and between men and women were examined using chi-square tests or independent *t*-tests depending on the nature of the dependent variable. Also, to analyze the distribution of BMI change in relation to the three SF-36 vitality categories, the tertiles of BMI change were calculated and used as a categorical variable in chi-square tests.

Preliminary analyses showed sex differences in several key variables including BMI and SF-36 vitality, and a significant interaction of sex and SF-36 vitality on the risk of developing obesity was observed (*p*=0.049). In line with previous studies, the main analyses were therefore stratified by sex. Also, cohort differences were found on all characteristics. Accordingly, cohort status was adjusted for in all the conducted analyses.

Changes in BMI from baseline to follow-up assessment were analyzed in four models: Model 1 adjusted for cohort, age, and baseline BMI; Model 2 adjusted for cohort, age, baseline BMI, and level of education; Model 3 adjusted for cohort, age, baseline BMI, smoking, and level of physical activity; Model 4 adjusted for cohort, age, baseline BMI, and obesity-related diseases including type 2 diabetes, hypertension, ischemic heart disease, and other hypothyroidism. Analyses of BMI were conducted using linear regression analyses and are shown for three different populations. First, associations of SF-36 vitality with BMI change were investigated in the full sample. Secondly, as previous research has suggested that prospective associations of BMI and HRQOL differ between individuals gaining and losing weight [11], the associations were investigated separately for participants gaining weight and participants either maintaining or losing weight over the follow-up period.

The risk of becoming obese was analyzed in four models with the same covariates as described above. Participants with BMI ≥30 kg/m^2^ at baseline were excluded from these analyses, and logistic regression was applied to investigate associations of SF-36 vitality with obesity as outcome. Because preliminary analyses showed that no women in the high SF-36 vitality category were obese at follow-up, a binary SF-36 vitality variable was used in all analyses of obesity with the high and the medium categories merged.

## 4. Results

Table 1 presents participant characteristics including sex, age, BMI, obesity status, follow-up BMI, obesity status at follow-up, level of education, level of physical activity, smoking, prevalence of obesity-related diseases, and the distribution of the SF-36 vitality categories separately for the two baseline cohorts. By design, the two cohorts included in the study differ on sex as the MP consists of men only, while the DALWUH includes both men and women. The total sample of the present study, therefore, consists of 2864 (81.5%) men and 648 (18.5%) women with a mean age of 52 years. The mean baseline BMI was 26.0 kg/m^2^ and 24.6 kg/m^2^ for men and women, respectively, and 326 (11.4%) men and 62 (9.6%) women were categorized as obese at baseline. Of the total 3512 participants in the current study, almost 40% reported to have high educational level and 30% reported a high physical activity level. However, only 19.4% of the participants were categorized as high on SF-36 vitality and 37.5% fell in the low SF-36 vitality category.

The tertiles of BMI change and the chi-square tests of SF-36 vitality with the tertiles of BMI change are shown for men and women in Table 2. Among both men and women, those scoring high on SF-36 vitality were most likely to have a small weight gain corresponding to the second tertile of BMI change. However, these associations were only significant among women (*p*=0.002).

Associations of SF-36 vitality with BMI are shown in Table 3 for the full sample and for the weight gain group and the weight loss group, respectively. For the *full sample*, SF-36 vitality was significantly associated with BMI change among men independently of cohort, age, baseline BMI, level of education, and obesity-related diseases. A trend was observed when adjusting for level of physical activity and smoking in addition to cohort, age, and baseline BMI. Men scoring low on SF-36 vitality had a 0.05–0.07 (*p* < 0.05) standard deviation larger BMI increase than high-scoring men depending on the investigated model. SF-36 vitality did not predict BMI change in any of the investigated models among women.

Of the total sample, 2377 participants gained weight over the follow-up period while 1135 maintained or lost weight with a mean BMI increase of 1.4 (SD: 1.3) and a mean BMI decrease of 1.1 (SD: 1.1) in the two groups, respectively. In the *weight gain group*, significant associations of SF-36 vitality were found for both men and women. Among men, the low SF-36 vitality group had a significantly higher BMI increase over the follow-up period compared with the high SF-36 vitality group (*p* < 0.001 in all models). BMI increases were 0.09–0.13 standard deviations larger among men with low baseline SF-36 vitality compared with men in the high vitality category. Men in the medium SF-36 vitality category did not differ significantly from their high-scoring counterparts with regards to BMI changes from baseline to follow-up. Among women, SF-36 vitality also predicted BMI increases in all models. However, women in the medium and low SF-36 vitality category did not differ significantly from the high-scoring women possibly reflecting the relatively small number of women in the current study.

In the *weight maintenance/weight loss group*, SF-36 vitality at baseline was not associated with BMI change among men or women in any of the investigated models.

Table 4 presents associations of baseline SF-36 vitality with obesity status at follow-up in men and women. SF-36 vitality did not predict obesity at follow-up among men. In contrast, among women, SF-36 vitality predicted obesity at follow-up in all the investigated models with women in the low SF-36 vitality category having 6.3 (*p*=0.006) times higher odds of becoming obese at follow-up than women in the high SF-36 vitality group. Furthermore, this association remained significant when adjusting for level of education, level of physical activity, smoking, and obesity-related diseases in addition to cohort, age, and baseline BMI (odds ratios ranging from 5.8–7.1; *p* < 0.05). The previously described significant interaction of sex and SF-36 vitality on the risk of becoming obese (*p*=0.049) indicates that the different results for men and women were statistically significant.

## 5. Discussion

To our knowledge, this is one of the first studies to investigate the effect of SF-36 vitality on BMI changes and the development of obesity. Results indicated that SF-36 vitality was of predictive value for changes in BMI among men and women gaining weight over the five-year follow-up period with larger increases in BMI observed in individuals with low SF-36 vitality at baseline compared with individuals reporting high baseline SF-36 vitality. In contrast, SF-36 vitality was not related to changes in BMI among men and women losing weight. Furthermore, SF-36 vitality was significantly associated with development of obesity among women.

The average BMI change of men and women in the current study was modest (0.62 (SD: 1.7) BMI units for men and 0.64 (SD: 1.8) for women). The results in Table 2 revealed that most women in the high SF-36 vitality group fell in the second tertile corresponding to a minor BMI increase of 0.4–1.1 BMI units. This is in line with previous research showing that people in developed countries typically gain weight until they are about 60 years old [24]. Furthermore, there is consistent evidence that excess weight does not constitute the same health risk among middle-aged and elderly individuals as it does among younger individuals, but may in fact be protective of age-related health problems [25]. Thus, studies emphasize a distinction between smaller weight gains that may be expected in certain age groups and greater weight gains, which may reflect underlying pathological causes, resulting in obesity.

### 5.1. Associations of SF-36 Vitality with BMI Change and Obesity

There is convincing evidence that HRQOL varies with BMI, and further that HRQOL is substantially reduced among obese individuals [10, 26]. However, while longitudinal studies have recognized the predictive value of BMI and obesity on HRQOL, research that prospectively has investigated the reverse association, the influence of HRQOL on BMI and/or obesity, is extremely scarce. In the current study, we found that SF-36 vitality is not merely a consequence of weight gain or obesity but may also be regarded as a predictor of weight status over time. Interestingly, SF-36 vitality was only of predictive value for BMI changes among individuals with increasing BMI during the follow-up period. These results are in line with the only previous study prospectively investigating the effect of HRQOL on obesity [11]. In their study, Cameron et al. [11] followed 5985 Australian men and women with a mean age of 51.3 years over a five-year follow-up period and found that the mental components of HRQOL including the SF-36 vitality scale predicted weight gain over the follow-up period. More specifically, individuals in the lowest baseline SF-36 vitality tertile gained more BMI units than individuals in the medium and high SF-36 vitality tertiles though the exact estimates were not reported. However, findings from both the current study and the Cameron et al. [11] study indicate that there is no clear dose-dependent relationship between SF-36 vitality and increases in BMI as only individuals with the lowest baseline vitality had significantly greater increases in BMI.

### 5.2. Possible Underlying Mechanisms

The SF-36 vitality scale is included in the mental component of the SF-36. However, studies have consistently reported moderate to strong correlations between the physical and mental components of HRQOL [27, 28], emphasizing the complex relation between physical and mental factors in HRQOL. Previous research has suggested that weight gain and obesity mainly are predictors of the physical components of HRQOL [12]. However, in line with the Cameron et al. [11] study who emphasized the effect of the mental component on weight gain, the current findings suggested that SF-36 vitality is of importance for increases in BMI and development of obesity. Thus, results of the current study further add to the evidence that different components of HRQOL are relevant for associations of HRQOL and weight status depending on the direction of this relation.

Several plausible mechanisms may link SF-36 vitality and weight gain. Reduced vitality may lead to increases in BMI through a lack of motivation, energy, and/or capacity to engage in physical activity. In fact, previous research has suggested that individuals with poor SF-36 vitality report significantly more sedentary activity compared with individuals with high SF-36 vitality [29]. However, in the current study, adjusting for level of physical activity did not influence the association between SF-36 vitality and changes in BMI or obesity substantially. Thus, our results are not supportive of this hypothesis. Furthermore, the correlation between SF-36 vitality at baseline and level of physical activity at follow-up was weak (*r* = 0.16), indicating that level of physical activity from baseline to follow-up does not influence the reported associations between SF-36 vitality and weight gain.

Possible biological explanations for the observed associations between SF-36 vitality and BMI change and development of obesity were not investigated in the present study. For instance, reduced SF-36 vitality may contribute to weight gain and obesity through stress-related systems including the hypothalamic-pituitary-adrenal axis [30]. Such biological pathways have been suggested to contribute to the relation between depression and increased BMI and obesity [31, 32], and it is possible that similar mechanisms are responsible for the associations between mental components of HRQOL and obesity found in prior research [11] and more specifically, between SF-36 vitality and weight status observed in the current study. Future studies examining the relation between HRQOL and weight status should include potential underlying biological mechanisms.

Lastly, psychological pathways related to eating behavior and body dissatisfaction may underlie associations of SF-36 vitality and obesity. Interestingly, in the current study, low SF-36 vitality predicted development of obesity among women in all the tested models with ORs ranging from 5.8 to 7.1 (*p* < 0.05) while this association was not significant among men. Thus, results of the current study indicate that sex differences may exist in the relation between SF-36 vitality and obesity. Research has consistently linked emotionally induced eating [33, 34] and body dissatisfaction with obesity [35] especially among women. Thus, the observed sex differences in the relation between SF-36 vitality and obesity may indicate that psychological mechanisms play a greater role in the development of obesity among women than among men. We did not observe substantial sex differences in the relation between SF-36 and BMI change, and it may be hypothesized that psychological mechanisms constitute a more important factor for the development of obesity than for variations within the nonobese BMI range.

### 5.3. Strengths and Limitations

This study has several strengths including a prospective study design, a large community-based sample, objectively measured weight and height at follow-up, detailed information about sociodemographic factors and physical activity, and register-based information about obesity-related medical diseases. Yet, some limitations should be mentioned.

The average BMI increase among weight gainers was 1.5 BMI points (SD: 1.3) for men and 1.4 (SD: 1.2) for women during the follow-up period, which must be considered moderate changes. However, the follow-up period of the current study was 3–7 years, and it may be that BMI changes become clinically relevant when they are considered over the years of a lifetime.

Also, baseline BMI was based on self-reported height and weight, while BMI at follow-up was physically measured at an examination. Considering the consistently found tendency to underreport weight [36], BMI changes in the current study may have been overestimated. However, the mean measured and self-reported BMI at follow-up did not differ (26.33 kg/m^2^ versus 26.33 kg/m^2^), indicating that participants in the current study are not prone to underreport their weight.

The availability of extensive data from two large community cohorts is an advantage of the current study. However, by design, there is very little age variation within the cohorts. Age has previously been found to be one of the most consistent predictors of overweight and obesity with middle-aged and older individuals at higher risk of being obese than their younger counterparts [37, 38]. Members of the included cohorts were all either 47, 51, or 57 years of age at baseline, and thus results of the present study are only relevant for middle-aged individuals. Furthermore, cultural differences may affect public and individual perspectives on obesity [39], and it is therefore uncertain whether the current results can be generalized beyond the Danish population. Furthermore, the CAMB had a relatively modest response rate of 30%, which should be considered when interpreting the results. For instance, it is likely that participants and nonparticipants in the CAMB study differ on individual characteristics such as level of education and general health [23] with only the more resourceful individuals participating in the follow-up study. That is, the effect of SF-36 vitality on weight status may possibly have been underestimated in the current study.

Lastly, as discussed earlier, we did not investigate the effect of biological mechanisms on the SF-36 vitality-BMI change/obesity relation. Likewise, the current study focused on the vitality scale, which is encompassed in the mental component of the SF-36. However, it would have been interesting to also examine the predictive value of other SF-36 scales, especially to include subscales from the physical SF-36 component. Unfortunately, this information was not available in the current study.

## 6. Conclusion

The study findings indicate that the SF-36 vitality scale is of predictive value for increases in BMI over time among individuals gaining weight, but not among individuals maintaining or losing weight. Furthermore, SF-36 vitality seems to be of importance for the development of obesity among women. Thus, it seems that some sex differences exist in the relation between SF-36 vitality and obesity possibly related to previously reported sex differences in the psychological correlates of obesity. It is plausible that different mechanisms underlie variations within the nonobese BMI range and variations in obesity emphasizing the importance of distinguishing between smaller weight changes and larger increases leading to obesity. Nevertheless, the identification of poor SF-36 vitality as a potential risk indicator for weight gain and development of obesity may be beneficial in clinical practice.

## Figures and Tables

**Table 1:** Characteristics of participants in the Metropolit 1953 Danish Male Birth Cohort, the Danish Longitudinal Study on Work, Unemployment and Health, and the total study sample.

	MP	DALWUH	*p* value^1^
*N*	2371	1141	—

*Sex (%)*			*0.000* (by design)
Men	100.0	42.7	
Women	0	57.3	

*Age*, *M (SD)*	51.0 (0.2)	52.8 (5.0)	*0.000* (by design)

*Level of education (%)*			0.070
High	39.4	41.0	
Medium	47.3	48.5	
Low	13.3	10.5	

*BMI at baseline*, *M (SD)*	25.9 (3.8)	25.4 (4.2)	*0.000*

*Obesity baseline (%)*			0.304
Yes	10.7	11.8	
No	89.3	88.2	

*BMI at follow-up*, *M (SD)*	26.3 (3.8)	25.9 (4.4)	*0.000*

*Obesity follow-up (%)*			0.166
Yes	15.8	14.0	
No	85.2	86.0	

*Physical activity (%)*			*0.000*
High	27.3	34.6	
Medium	58.5	56.5	
Low	14.2	8.9	

*Smoking (%)*			*0.000*
Yes	34.0	25.0	
No	66.0	75.0	

*Obesity-related diseases (%)*			
Type 2 diabetes	1.6	2.1	*0.039*
Hypertension	3.5	3.7	0.100
Ischemic heart disease	3.9	3.2	0.829
Hypothyroidism	0.2	0.7	*0.002*

*SF-36 vitality*, *M (SD)*	68.0 (18.9)	62.0 (19.7)	*0.000*

*SF-36 vitality (%)*			*0.000*
High	21.8	14.5	
Medium	45.2	38.4	
Low	32.0	47.1	

^1^ *p* value of the chi-square test or independent *t*-test of differences between the means—categorical and continuous outcomes, respectively. SD, standard deviation; M,  mean; MP, the Metropolit 1953 Danish Male Birth Cohort; DALWUH, the Danish Longitudinal Study on Work, Unemployment and Health.

**Table 2:** Tertiles of change in BMI for men and women by SF-36 vitality category. *p* values for chi-square tests are presented.

	Change in BMI (tertiles)		
*Men*			
SF-36 vitality	−9.4–0 BMI units	0.1–1.1 BMI units	1.2–11.0 BMI units
High (%)	35	35	30
Medium (%)	34	35	31
Low (%)	33	32	35
*p* value^1^			0.126

*Women*			
SF-36 vitality	−13.7–0.3 BMI units	0.4–1.1 BMI units	1.2–8.5 BMI units
High (%)	26	43	31
Medium (%)	42	32	26
Low (%)	37	26	37
*p* value^1^			**0.002**

^1^ *p* value for the chi-square test of association between SF-36 category and the tertiles of change in BMI.

**Table 3:** Linear regression (standardized beta) of SF-36 vitality on BMI change among men and women. Full sample and separate for men and women gaining and maintaining/losing weight.

	Model 1^a^		Model 2^b^		Model 3^c^		Model 4^d^		Model 1^a^		Model 2^b^		Model 3^c^		Model 4^d^	
	*β*	*p* ^1^	*β*	*p* ^1^	*β*	*p* ^1^	*β*	*p* ^1^	*β*	*p* ^1^	*β*	*p* ^1^	*β*	*p* ^1^	*β*	*p* ^1^
*Full sample*	Men (*N*=2864)								Women (*N*=648)							
SF-36 vitality		**0.011**		**0.036**		0.062		**0.022**		0.159		0.182		0.241		0.112
High	—		—		—		—		—		—		—		—	
Medium	0.02		0.02		0.01		0.02		−0.08		−0.08		−0.07		−0.08	
Low	0.07^*∗*^		0.06^*∗*^		0.05^*∗*^		0.06^*∗*^		0.01		0.0		0.01		0.02	

*Weight gain*	Men (*N*=1920)								Women (*N*=457)							
SF-36 vitality		**<0.001**		**<0.001**		**0.001**		**<0.001**		**<0.001**		**0.001**		**0.002**		**<0.001**
High	—		—		—		—		—		—		—		—	
Medium	0.03		0.02		0.02		0.03		−0.02		−0.03		−0.04		−0.03	
Low	0.13^*∗∗*^		0.12^*∗∗*^		0.09^*∗*^		0.13^*∗∗*^		0.12		0.11		0.11		0.13	

*Weight loss*	Men (*N*=944)								Women (*N*=191)							
SF-36 vitality		0.139		0.143		0.294		0.114		0.523		0.613		0.661		0.523
High	—		—		—		—		—		—		—		—	
Medium	−0.03		−0.03		−0.03		−0.03		0.00		0.00		0.03		0.03	
Low	−0.08		−0.07		−0.06		−0.08		−0.03		−0.01		0.01		0.00	

^a^Adjusted for cohort, age, and baseline BMI. ^b^Adjusted for cohort, age, baseline BMI, and education. ^c^Adjusted for cohort, age, baseline BMI, physical activity, and smoking. ^d^Adjusted for cohort, age, baseline BMI, and obesity-related diseases. ^1^*p* value of the *F*-test. ^*∗*^Significantly different from reference value at *p* < 0.05. ^*∗∗*^Significantly different from reference value at *p* < 0.001.

**Table 4:** Logistic regression of associations between SF-36 vitality (high/low) and obesity (BMI ≥30 kg/m^2^) among men and women.

	Men (*N*=2543)								Women (*N*=586)							
	Model 1^a^		Model 2^b^		Model 3^c^		Model 4^d^		Model 1^a^		Model 2^b^		Model 3^c^		Model 4^d^	
	OR (95% CI)	*p* ^1^	OR (95% CI)	*p* ^1^	OR (95% CI)	*p* ^1^	OR (95% CI)	*p* ^1^	OR (95% CI)	*p* ^1^	OR (95% CI)	*p* ^1^	OR (95% CI)	*p* ^1^	OR (95% CI)	*p* ^1^
SF-36 vitality		0.253		0.255		0.560		0.271		**0.006**		**0.010**		**0.005**		**0.008**
High	—		—		—		—		—		—		—		—	
Low	1.2 (0.9; 1.8)		1.2 (0.9; 1.8)		1.1 (0.8; 1.6)		1.2 (0.9; 1.8)		6.3 (1.7; 23.8)^*∗*^		5.8 (1.5; 21.9)^*∗*^		7.1 (1.8; 28.2)^*∗*^		6.0 (1.6; 22.4)^*∗*^	

^a^Adjusted for cohort, age, and baseline BMI. ^b^Adjusted for cohort, age, baseline BMI, and education. ^c^Adjusted for cohort, age, baseline BMI, physical activity, and smoking. ^d^Adjusted for cohort, age, baseline BMI, and obesity-related diseases. ^1^*p* value of the *F*-test. ^*∗*^Significantly different from reference value at *p* < 0.05. ^*∗∗*^Significantly different from reference value at *p* < 0.001. CI, confidence interval.
